# Supplementary material for: PBP Target Profiling by β-Lactam and β-Lactamase Inhibitors in Intact Pseudomonas aeruginosa: Effects of the Intrinsic and Acquired Resistance Determinants on the Periplasmic Drug Availability
Source: Microbiol Spectr. 2022 Dec 8;11(1):e03038-22. doi: 10.1128/spectrum.03038-22 (PMC9927461; doi:10.1128/spectrum.03038-22)
Supplement: Supplemental file 1 — Supplemental material. Download spectrum.03038-22-s0001.pdf, PDF file, 0.5 MB [file spectrum.03038-22-s0001.pdf]

SUPPLEMENTARY DATA.

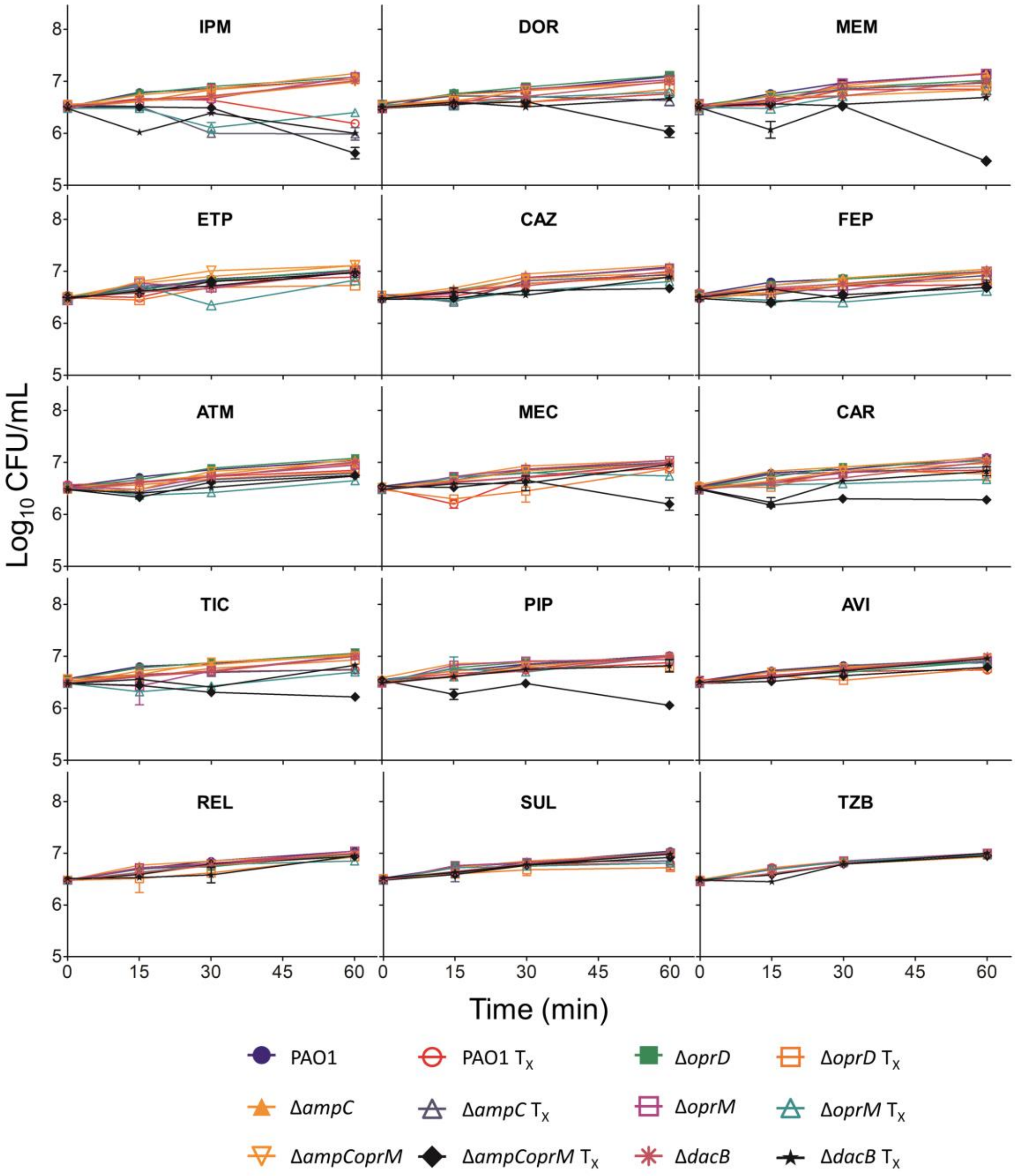

Figure S1. Time kill curves of *P. aeruginosa* PAO1 and isogenic mutants PAΔ*oprD*, PAΔ*ampC*, PAΔ*oprM*, PAΔ*ampCoprM and* PAΔ*dacB*. Cultures were grown (initial inoculum = $10^{6.5}$ CFU/mL) in MH or treated (T_X) for 15, 30 and 60 min with 1/2 × MIC imipenem (IPM), doripenem (DOR), meropenem (MEM), ertapenem (ETP), ceftazidime (CAZ), cefepime (FEP), aztreonam (ATM), mecillinam (MEC), carbenicillin (CAR), ticarcillin (TIC), piperacillin (PIP), avibactam (AVI), relebactam (REL), sulbactam (SUL) or tazobactam (TZB). The mean values from at least three experiments ± standard deviations are shown.

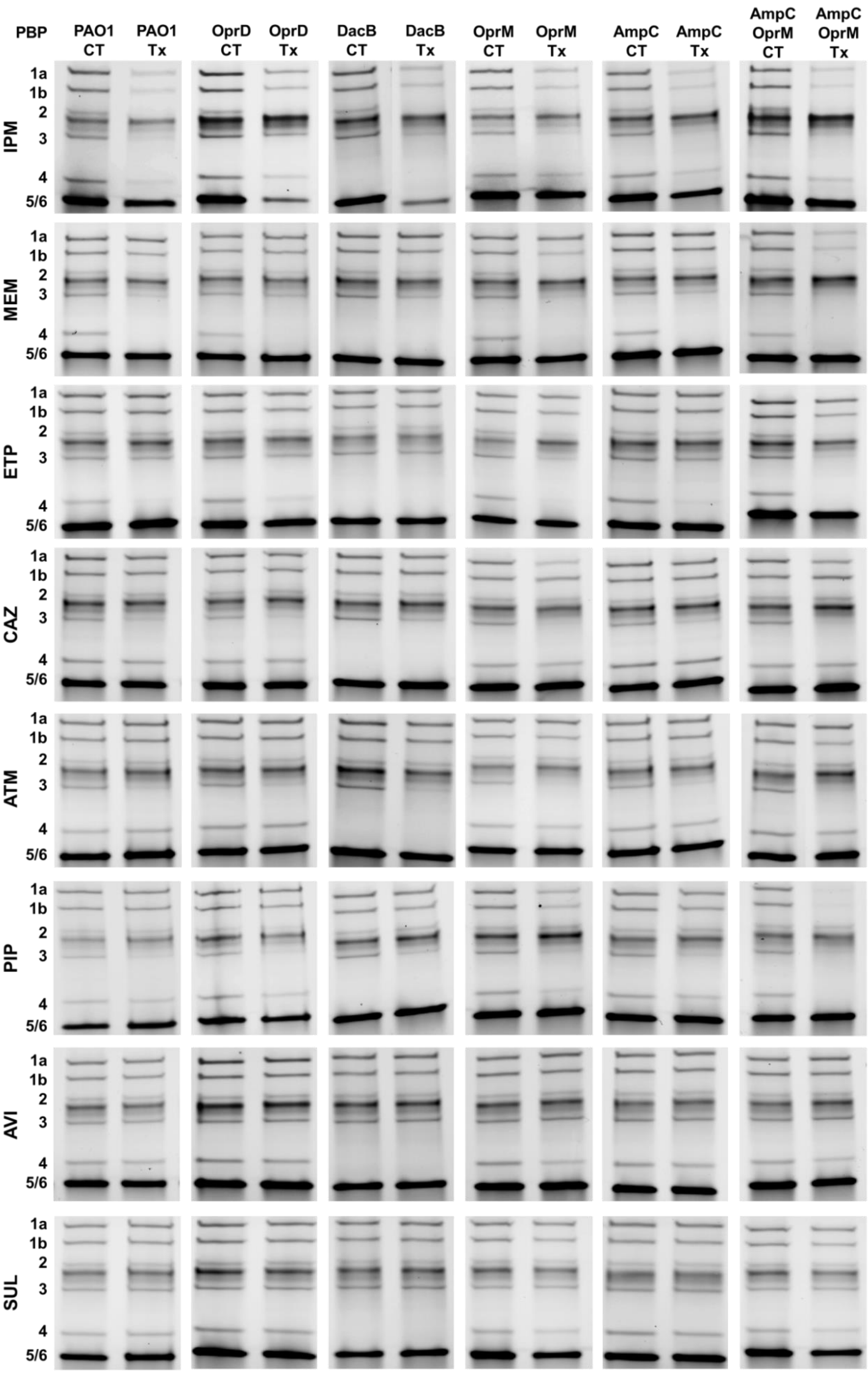

Figure S2. Representative example of the whole cell PBP-binding assay gels. *P. aeruginosa* PAO1 and isogenic mutants cultures were incubated (Tx) for 30 min in the presence of imipenem (IPM), meropenem (MEM), ertapenem (ETP),

ceftazidime (CAZ), aztreonam (ATM), piperacillin (PIP), avibactam (AVI) and sulbactam (SUL). Resulting 0.5 mg/mL of antibiotic-bound PBP-containing membrane preparations were labeled with 25 μM bocillin FL. Antibiotic concentrations used were (PAO1 1/2 × MIC): IPM = 0.5 µg/mL; MEM = 0.25 µg/mL; ETP = 4 µg/mL; CAZ = 0.5 µg/mL; ATM = 2 µg/mL; PIP = 2 µg/mL. BLIs AVI and SUL were dosed at a fixed concentration of 4 µg/mL. Labeled PBPs were separated through SDS-polyacrylamide gels and visualized using a fluorimager (excitation at 488 nm and emission at 530 nm).

Table S1. Dissociation and Bocillin FL displacement studies.

Drug	PBP	Bocillin FL labelling protocol[a]							
		Standard		15 min pre-isolation		5 min post-isolation		30 min post-isolation	
		MEAN	SD	MEAN	SD	MEAN	SD	MEAN	SD
IPM	PBP1a	0.72	0.06	0.70	0.06	0.76	0.09	0.79	0.02
	PBP1b	0.73	0.08	0.64	0.09	0.79	0.12	0.81	0.08
	PBP2	0.73	0.12	0.80	0.14	0.83	0.07	0.68	0.08
	PBP3	0.62	0.15	0.71	0.11	0.73	0.09	0.75	0.11
	PBP4	0.74	0.04	0.68	0.08	0.80	0.06	0.69	0.04
	PBP5/6	0.67	0.06	0.59	0.09	0.61	0.07	0.66	0.06
CAZ	PBP1a	0.22	0.04	0.25	0.07	0.29	0.11	0.27	0.06
	PBP1b	0.12	0.01	0.12	0.04	0.16	0.04	0.12	0.01
	PBP2	0.12	0.17	0.06	0.11	0.02	0.07	0.08	0.13
	PBP3	0.38	0.05	0.41	0.07	0.59	0.15	0.54	0.11
	PBP4	0.13	0.10	0.20	0.12	0.13	0.07	0.19	0.10
	PBP5/6	0.02	0.09	0.08	0.06	0.04	0.04	0.04	0.09
REL	PBP1a	0.08	0.22	0.06	0.16	0.13	0.16	0.09	0.16
	PBP1b	0.09	0.24	0.16	0.11	0.15	0.15	0.15	0.22
	PBP2	0.11	0.25	0.13	0.18	0.09	0.09	0.16	0.17
	PBP3	0.09	0.08	0.02	0.07	0.12	0.11	0.05	0.07
	PBP4	0.18	0.11	0.14	0.09	0.30	0.19	0.20	0.11
	PBP5/6	0.13	0.33	0.02	0.10	0.15	0.18	0.05	0.08

[a] To assess possible drug-PBP dissociation during the membrane isolation or drug displacement by Bocillin FL we compared the PBP binding profiles of three drugs (IPM, imipenem; CAZ, ceftazidime and REL, relebactam) under several experimental conditions in the wild type PAO1 strain. After the 30 min drug incubation, we labeled whole cells by incubating them with Bocillin FL for 15 min before sonication and membrane isolation and compared the binding results with the standard procedure (Bocillin FL labelling for 15 min after membranes isolation). To analyze drug displacement by Bocillin FL after membranes isolation, different incubation times were studied (5 and 30 min) and compared with the standard 15 min incubation. No significant differences were found. We

selected the 15 min post- isolation of membranes labeling for our experiments because it was the most efficient experimental setting.

TABLE S2. PBP binding (IC_{50}) of β-lactam antibiotics and BLIs in lysed *P. aeruginosa* PAO1.

PBP	IC_{50} of the indicated drug (mg/L)[a]									
	IPM[b]	DOR	MEM[c]	ETP	CAZ	FEP[c]	ATM	MEC	PIP	AVI[b]
1a	0.19	0.43	0.26	0.35	**0.16**	**0.12**	2.11	>4	1.57	>32
1b	0.13	0.37	0.21	0.36	>4	0.82	2.95	>4	1.11	3.6
2	**0.05**	**0.06**	**0.13**	**0.04**	>4	2.71	>4	**0.19**	3.17	4.2
3	0.34	**0.13**	**0.06**	**0.19**	**0.06**	**0.05**	**0.08**	>4	**0.05**	>32
4	**0.01**	**0.01**	**0.01**	**0.02**	2.15	2.52	>4	>4	1.45	3.1
5/6	0.52	1.41	0.31	2.21	>4	>4	>4	>4	>4	2.2

[a] Concentration of β-lactam that inhibits 50 % of Bocillin FL compared to no drug control. IPM, imipenem; DOR, doripenem; MEM, meropenem; ETP, ertapenem; CAZ, ceftazidime; FEP, cefepime; ATM, aztreonam; MEC, mecillinam; PIP, piperacillin; AVI, avibactam. Bold numbers represent the selective PBPs for a given compound (i.e. $IC_{50} \geq$ 4-fold lower than that of the next most inhibited PBP) [1].

[b] IPM and AVI IC_{50} values reproduced from our previous work Lopez-Argüello et al, 2021 [2].

[c] MEM and FEP IC_{50} values reproduced from our previous work Moya et al, 2017 [3].

REFERENCES

1. Kocaoglu, O. and E.E. Carlson, *Profiling of beta-lactam selectivity for penicillin-binding proteins in Escherichia coli strain DC2.* Antimicrob Agents Chemother, 2015. **59**(5): p. 2785-90.
2. López-Argüello, S., et al., *Molecular Basis of AmpC β-Lactamase Induction by Avibactam in Pseudomonas aeruginosa: PBP Occupancy, Live Cell Binding Dynamics and Impact on Resistant Clinical Isolates Harboring PDC-X Variants.* Int J Mol Sci, 2021. **22**(4): p. 3051.
3. Moya, B., et al., *WCK 5107 (Zidebactam) and WCK 5153 Are Novel Inhibitors of PBP2 Showing Potent "β-Lactam Enhancer" Activity against Pseudomonas aeruginosa, Including Multidrug-Resistant Metallo-β-Lactamase-Producing High-Risk Clones.* Antimicrob Agents Chemother, 2017. **61**(6).
